# Promising Molecular Targets in Pharmacological Therapy for Neuronal Damage in Brain Injury

**DOI:** 10.3390/antiox12010118

**Published:** 2023-01-03

**Authors:** Cristóbal de los Ríos, Lucía Viejo, Victoria Jiménez Carretero, Natalia Hernández Juárez, Natália Cruz-Martins, Jesús M. Hernández-Guijo

**Affiliations:** 1 Department of Pharmacology and Therapeutic and Teófilo Hernando Institute, Faculty of Medicine, University Autónoma de Madrid, C/. Arzobispo Morcillo 4, 28029 Madrid, Spain; 2 Departamento de Ciencias Básicas de la Salud, University Rey Juan Carlos, Avda. Atenas s/n, 28922 Alcorcón, Spain; 3 Faculty of Medicine, Institute for Research and Innovation in Health (i3S), University of Porto, 4200-319 Porto, Portugal; 4 Institute for Research and Advanced Training in Health Sciences and Technologies, Rua Central de Gandra, 1317, 4585-116 Gandra, Portugal; 5 Ramón y Cajal Institute for Health Research, IRYCIS, Hospital Ramón y Cajal, Ctra. de Colmenar Viejo, Km. 9,100, 28029 Madrid, Spain

**Keywords:** brain injury, neurodegenerative diseases, protein phosphatase 2A, PP2A-activating drugs, mitochondrial calcium, benzothiazepines

## Abstract

The complex etiopathogenesis of brain injury associated with neurodegeneration has sparked a lot of studies in the last century. These clinical situations are incurable, and the currently available therapies merely act on symptoms or slow down the course of the diseases. Effective methods are being sought with an intent to modify the disease, directly acting on the properly studied targets, as well as to contribute to the development of effective therapeutic strategies, opening the possibility of refocusing on drug development for disease management. In this sense, this review discusses the available evidence for mitochondrial dysfunction induced by Ca^2+^ miscommunication in neurons, as well as how targeting phosphorylation events may be used to modulate protein phosphatase 2A (PP2A) activity in the treatment of neuronal damage. Ca^2+^ tends to be the catalyst for mitochondrial dysfunction, contributing to the synaptic deficiency seen in brain injury. Additionally, emerging data have shown that PP2A-activating drugs (PADs) suppress inflammatory responses by inhibiting different signaling pathways, indicating that PADs may be beneficial for the management of neuronal damage. In addition, a few bioactive compounds have also triggered the activation of PP2A-targeted drugs for this treatment, and clinical studies will help in the authentication of these compounds. If the safety profiles of PADs are proven to be satisfactory, there is a case to be made for starting clinical studies in the setting of neurological diseases as quickly as possible.

## 1. Introduction

Neuronal damage associated with brain injury and neurodegenerative diseases (ND) have an increasing incidence at worldwide level, and are currently conceived as having a permanent, incurable, and progressive nature, linked to a marked loss in neuronal activity [1,2]. The rate of neuronal death increases progressively over time, ultimately leading to a worsening of the clinical condition. Various ND are currently known, in addition to stroke, with amyotrophic lateral sclerosis (ALS), Alzheimer’s disease (AD), Huntington’s disease (HD), Parkinson’s disease (PD), and some types of dementia being the most common. These heterogeneous diseases are characterized by complex neurodegenerative processes, undergoing progressive deterioration in both the structure and function of central nervous system (CNS) and peripheral nervous system (PNS) [1,2,3]. Based on the pathological and clinical features, these pathologies can be classified into chronic and acute [4]. Chronic ND includes AD, PD, HD, and psychiatric disorders, whereas acute ND includes cerebral ischemia, epilepsy, and brain injury [3], differing in their pathophysiology. Some cause cognitive and memory impairments and others trigger the inability to breathe, move, and speak, while also affecting daily activities, such as talking, thinking, balance, heart function, and other brain functions [5,6,7,8]. 

In the past two decades, extensive research has been conducted to understand the main triggering factors of the various and complicated pathways of neuronal damage associated with ND, which despite the advances stated so far are not too significant. Aging is conceived as the primary risk factor, despite of its multifactorial nature, involving protein misfolding, neurotoxic oligomers, mitochondrial dysfunction, protein modifications, protein deposition, oxidative stress, environmental factors, metal accumulation, and genetic factors. Over time, the list of triggering factors for these clinical situations is increasing, but the exact cascade of events that culminate with neuronal death has not yet been well understood. For almost every type, diverse hypotheses on the mechanisms responsible for neuronal dysfunction have been offered [9]. Nonetheless, with more than one hundred types of pathologies currently identified, most of them overlap each other at both pathological and clinical levels, making it hard to classify them in practical terms. 

The classification of these pathologies is usually based on: (1) clinical symptoms which are determined by the evaluation of the anatomic regions where neuronal dysfunction is stated; and (2) by detecting the biochemical modification and deposition of intracellular or extracellular proteins in glial cells or neurons. This clinical classification, besides other benefits, is very helpful for estimating the early symptoms and for clinical identification [10]. Regarding the two classification approaches, the following aspects need to be stressed: (1) Dementia, cognitive decline, and variations in brain functions involve anatomic structures such as the entorhinal cortex, neo-cortical areas, hippocampus, and limbic system. Frontotemporal dementia is linked to the dysfunction of the frontal and temporal lobes [10]; (2) Hyperkinetic and hypokinetic movement disorders, symptoms linked to cell dysfunction or the participation of lower and upper motor neurons, where the most important anatomic parts involved are the thalamus, lower motor neurons of the spinal cord, nuclei, brainstem nuclei, basal ganglia, and cerebellar cortex [10]. An important characteristic of many of these pathologies is the accumulation of intracellular and extracellular protein depositions [11]; among the most notable we can mention are: Tau (microtubule-associated protein) encoded by gene microtubule-associated protein tau (MAPT); amyloid beta (Aβ) peptide cleaved from amyloid precursor protein (APP); alpha-synuclein encoded by α-synuclein (SNCA) gene; prion protein encoded by prion protein (PRNP) gene; transactive response DNA binding protein-43 (TDP-43) encoded by gene TARDBP; and the FET protein family, which includes Ewing sarcoma DNA binding protein 1 (EWSR1), fused-in-sarcoma (FUS), and TATA-binding linked factor 15 (TAF15). 

## 2. Brain Injury and Neurodegenerative Diseases: An Overview

Strokes cause high mortality rates around the world, being one of the main factors of disability in adults. That is why it is a priority to seek new therapeutic strategies to treat these situations since the existing ones are very limited. A cerebrovascular accident is produced by an abrupt reduction in the supply of oxygen and nutrients, either due to hemodynamic failure due to a lack of irrigation or due to cerebral hemorrhage. It is estimated that if the blood flow decreases by more than 75% for a period of more than 30 min, the death of neurons and the glia occurs. This ischemic nucleus is surrounded by a zone of penumbra (twilight zone), with reduced blood flow, but enough to be able to recover if normal perfusion is re-established in less than six hours [12]. If not, those cells die, and the ischemic area expands. For this reason, the twilight zone is considered ideal to prevent the progression of cerebral infarction through therapeutic interventions.

There are numerous mechanisms that participate in the cellular damage produced during cerebral ischemia. Initially, the energy failure triggered by the deficit of oxygen and glucose alters the maintenance of the membrane potential, producing a depolarization that causes the opening of ion channels, the release of neurotransmitters and the alteration of the activity of the transporters [13]. Many observations have shown that the development of neurotoxicity in ischemia, hypoxia, infarction, and brain trauma is linked to an exacerbated increase in glutamate in the extracellular space [14]. Most of the glutamate found in the nucleus of the infarcted area comes from the lysis of cells in this area [15]. However, in the twilight zone, glutamate comes from the infarct zone, as well as from that released by neurons and glia through different mechanisms such as exocytosis [16], channels activated by increased cell volume [17], the reversal of glutamate transporters [18], and by the increased function of the transporter that exchanges cysteine for glutamate [19]. Once accumulated in the extracellular space, glutamate massively activates its receptors, which cause an increase in cytosolic calcium, this being one of the main causes that triggers cell damage [20]. However, another cause is the increase in cell volume (cytotoxic edema) caused by the excessive activation of glutamate receptors [21], which together with vasogenic edema (a consequence of the breakdown of the blood–brain barrier) participates in the cerebral edema that contributes negatively to tissue recovery during ischemia [22]. That is why the prevention of edema is crucial for the treatment of cerebral ischemia.

Studies carried out in patients affected by cerebral ischemia have made it clear that the increase in glutamate in the brain is critical for causing neuronal damage [23]. However, effective treatments to intervene in cerebral ischemic processes are very limited. Clinical trials using drugs that target glutamatergic receptors have shown that they are ineffective or cause undesirable adverse effects [14]. Therefore, it is necessary to improve the knowledge of the molecular mechanisms involved in this disease to design new therapeutic strategies. 

The loss of cell membrane integrity that occurs during cell death not only causes the uncontrolled release of glutamate into the extracellular space, but also of other substances that can have detrimental effects on still viable cells. There is evidence that shows that the volume of ischemic damage is proportional to the amount of amino acids, not exclusively neurotoxic, released during ischemia, although to date the underlying mechanism of action has not been demonstrated [24,25]. One of the first consequences of cerebral ischemia is the loss of synaptic transmission, which is usually reversible in the twilight zone [26]. In fact, in our laboratory, we have shown for the first time that the disappearance of synaptic transmission caused by a period of hypoxia becomes irreversible if the lack of oxygen is accompanied by the presence of certain non-excitatory amino acids [27,28,29].

Presently, the most common ND in seniors is AD, the major cause of dementia at a worldwide level and accounting for approximately 60–80% of all dementias [30]. AD is generally characterized by a progressive loss of nerve cells/neurons as well as cognitive and brain function [31]. According to the World Health Organization, more than 50% of the population in developing countries suffer from AD and it is expected to rise to 70% by 2025 [32]. It is mainly triggered by factors such as the continuous deposition of Aβ proteins around the neurons, resulting in the formation of plagues, the hyper-phosphorylation of the tau protein with the formation of neurofibrillary tangles, and a decrease in the level of the neurotransmitter acetylcholine [33]. 

Parkinson’s disease (PD) is another progressive ND after AD, which is caused by a loss of nigrostriatal dopamine neurons and an accumulation of the intracellular protein α-synuclein. The lack of dopamine in the basal ganglia results in classical Parkinson’s motor symptoms, e.g., rigidity, bradykinesia, tremor, and lateral postural instability, along with non-motor symptoms that are also present in PD, which become troublesome in the later stages of PD [34]. 

Huntington’s disease (HD) is characterized by motor, psychiatric, and cognitive disturbance. It is triggered by an autosomal dominantly inherited trinucleotide (CAG) repeat expansion in the gene huntingtin (HTT) on chromosome 4, and as a result, abnormal HTT protein or mutant HTT (mHTT) protein is produced [35]. It is estimated that around 10.6–13.7 individuals per million in the Western population are suffering from HD, while in Hong Kong, Taiwan, and Japan the prevalence rate is lesser, i.e., around 1–7 per million, and in South Africa, a lower incidence of HD is observed in black population compared to the white and mixed population [36]. 

Amyotrophic lateral sclerosis (ALS), is characterized as a multisystem ND, widely known as a pure motor neuron disease [37,38,39]. ALS usually comprises adult-onset focal muscle weakness (most commonly starting in limb and distal muscles) and wasting, which further tend to increase with disease progression. At the genetic level, more than 20 genes have been discovered to be associated with ALS progression. The five major genetic causes are hexa-nucleotide expansion in chromosome 9 open reading frame 72 and mutations in TANK-binding kinase-1, TAR DNA-binding protein 43 (TARDBP), and superoxide dismutase 1 (SOD1) [40]. ALS has a worldwide prevalence of 1.75–3 individuals per million/year and a prevalence of 10–12 individuals per million in Europe [41,42]. Moreover, it has also been reported that men are at higher risk to develop ALS than women [43].

The effect of socioeconomic status on old-age dementia was also evaluated by Haselgren and colleagues. More specifically, they investigated the exposure of a few work environments, closely linked to socioeconomic status, on dementia with APOEε4 allele [44]. Data obtained in this study suggest that work control is the most significant benchmark in lowering the effect of APOEε4 allele on dementia; however, it is more significant in men than women. In another study, Toth et al. (2018) quantified the effect of socioeconomic factors (unemployment rate, gross domestic product (GDP) per capita, average wage and education, life expectancy) on the death rate of AD in the Slovak Republic between the years 2001–2015. Ordinary least squares were applied to conduct the study. According to the results, GDP, education, and average wage influence the mortality caused by AD [45]. In general, these studies suggest that socioeconomic status and occupation are linked to increased risk of ND. 

## 3. Cell Calcium Signal and the Role of Mitochondria

Calcium, one of the most abundant elements in nature is available in cells as divalent cation in different forms (complexed to organic compounds or bound to inorganic molecules) from the very beginning [46,47]. It has an enormous role in maintaining cellular physiology and biochemistry. Extracellular calcium contributes to bone formation and regulates the potential difference across the excitable cell membranes. However, cytoplasmic or intracellular calcium ion (Ca^2+^) concentration regulates different cellular functions, such as metabolism, secretion, fertilization, gene expression, proliferation and neural excitation [48,49,50]. The endoplasmic reticulum, mitochondria, and lysosomes have a key role in regulating calcium homeostasis [51]. Cytoplasmic Ca^2+^ ions also play an important role in signal transduction and cell-signaling pathways, where they act as important intracellular or second messengers. Additionally, Ca^2+^ is ideal for the messenger role due to its chemical properties and because it has a very high free energy concentration gradient, of about four orders of magnitude among extracellular and intracellular space [52,53]. 

Normally, the cytoplasmic Ca^2+^ concentration is around 100 nM, which is around 10,000-fold lower than the extracellular Ca^2+^ concentration [54], whereas a continuous and excessive increase in the cytosolic free calcium ([Ca^2+^]_c_) can cause the dysregulation of intracellular Ca^2+^ homeostasis [55], which results in cell death through apoptosis [56]. To stabilize the cytoplasmic concentration of Ca^2+^, it is transported from the cytosol to the extracellular fluid or to the ER and sometimes to mitochondria, along with those certain binding proteins (CBP) present in the organelles or cytoplasm that act as a Ca^2+^ buffer by binding with an excess of free Ca^2+^. Several researchers reported about the role of Ca^2+^ signaling in different ND, including AD [43], PD [57,58,59,60,61], HD [57,62,63], ALS [57,62,63,64,65], and autism spectrum disorders [66], among others. Thus, an increasing number of studies have assessed the dysregulation of Ca^2+^ signaling and its role in the development of ND, as well as in common aging [67,68,69,70,71,72,73].

The concept of Ca^2+^ signaling was first described by Prof. Sydney Ringer at University College, London, in 1883, who observed that the isolated heart could only be made to contract when Ca^2+^ was added to the perfusion medium, acting as a carrier signal [74]. After a long time, the same experiment was repeated by Heilbrunn and Wiercinski, (1947) who observed the contraction of frog muscles after a Ca^2+^ injection, while no activity was recorded with a Na^+^, K^+^, and Mg^2+^ injection [75]. The process of Ca^2+^ signaling consists of a sequence of biophysical and molecular incidents that are linked to external stimuli that trigger a specific intracellular response by increasing cytoplasmic [Ca^2+^]_c_ as a signal. 

Neurotransmitters, growth factors, or hormones are the most common external stimuli for Ca^2+^ signaling, although, in excitable cells, the initial chemical stimulus can result in membrane excitability, which in turn also stimulates the Ca^2+^-signaling pathway. Specialized channels and pumps are necessary for the proper functioning and mobilization of Ca^2+^ in cytoplasmic and extracellular spaces. It must be stressed that the likelihood of a high Ca^2+^ micro-domain increases drastically with concomitance in space and time of the activation of several Ca^2+^ channels on action potential firing. This localized Ca^2+^ is favored by the Ca^2+^-induced Ca^2+^ release (CICR) from the ER, through both inositol triphosphate (InsP3R) and ryanodine (RyR) receptors [76]. Moreover, the Ca^2+^ micro-domain formation is also activated by the spatial accumulation of the nucleus, mitochondria, ER, dendritic spikes or secretory vesicles [77,78,79,80,81]. Therefore, the Ca^2+^-signaling pathway is categorized by stimulating the generation of abundant [Ca^2+^]_c_ micro-domains that enable the regulation of various functions, using the same triggering signal but different time patterns and subcellular locations. In normal excitable cells, Ca^2+^ homeostasis is attained by fluxes occurring between three compartments, namely extracellular space, cytosol, and Ca^2+^ storing organelles. At the resting phase, the exchange rate of Ca^2+^ at the plasmalemma and ER membrane ranges from 1–10 µmol/l cells/s, whereas Ca^2+^ uptake through the mitochondrial Ca^2+^ uniporter (MCU) is extremely slow due to its exponential kinetics and poor Ca^2+^ affinity. However, at homeostasis, the [Ca^2+^]_c_ content is around 10^−7^ M in the mitochondrial matrix and cytosol, and 10^−3^ M at the ER lumen and extracellular space, so a very high gradient (104-fold) is required for the diffusion of Ca^2+^ in the cytosolic region [53]. Intracellular Ca^2+^ are stored in sub-organelles, such as mitochondria, endoplasmic/sarcoplasmic reticulum (ER/SR), nucleus, Golgi apparatus, and lysosomes [82], whereas mitochondria have a major role in cytosolic Ca^2+^ signaling as Ca^2+^ sensors/buffers. Thus, maintaining the fluxes (influx/efflux) through channels/pumps (MCU, the Na^+^/Ca^2+^ exchanger, etc.) and proteins [mitochondrial Ca^2+^ uptake protein family (MICU): 1, 2, etc.], they regulate Ca^2+^ homeostasis in cells [52]. At high stimulation rates, the [Ca^2+^]_c_ level increases, activating Ca^2+^ transport through the MCU, and, in that case, most of Ca^2+^ load is taken up by subcellular organelle mitochondria [83,84,85,86]. Then, with the termination of stimulation phase, the Ca^2+^ stored in mitochondria is effluxed to the cytosol within a few seconds or minutes [84,85]. In this way, the [Ca^2+^]_c_ level remains very high during this process, helping to mobilize secretory vesicles from the reserve pool to the membrane and thus to be ready for the next exocytotic events [87]. Mitochondria help in providing the extra energy required for the exocytotic release of neurotransmitters under intense stimulation and to resolve the Ca^2+^ load, thus maintaining cell homeostasis, whereas the Ca^2+^ deposited in the mitochondria helps in cell respiration and ATP synthesis [88,89]. 

Thus, mitochondrial damage may dysregulate Ca^2+^ signaling and affect its uptake, initiating cell death through apoptosis or necrosis by activating mitochondrial Ca^2+^ dependent disruptions, including mitochondrial Ca^2+^ buffering, mitochondrial dynamics (fusion, fission, trafficking) and mitophagy, and causing excitatory neurotoxicity, aging, ischemia-reperfusion in stroke and ND, such as AD, PD, and HD, among others.

## 4. Drugs Acting on Mitochondrial Calcium: The 4,1-benzothiazepines

The role of mitochondria in the regulation of the Ca^2+^ cycle has received much attention, as it is a central stage in neuronal survival and cell death. The benzothiazepine CGP37157 is a widely accepted molecule to explore and investigate the role of mitochondria in cell Ca^2+^ signaling through its blocking effects on the mitochondrial Na^+^/ Ca^2+^ exchanger (NCLX). The pharmacological actions of CGP37157 and its role as a NCLX blocker have been reported by different research groups using different in vitro and in vivo models, such as rabbit heart and rat brain mitochondria [90], guinea-pig heart and liver mitochondria [91], guinea-pig heart mitochondria [92], rabbit heart mitochondria [93], rat liver mitochondria [94], cultured rat ganglion neurons [95], cultured rat brain neurons [96], INS-1pancreatic beta cells [97], rat pancreatic islets [98], and rat atrial myocytes [99]. 

Nicolau and co-workers evaluated the cytoprotective effects of CGP37157 against veratridine-elicited chromaffin cell death. In this study, veratridine led to a concentration-dependent cell death that was nicely counteracted by CGP37157 (EC50 about 10 µM). Different events were significantly inhibited by CGP37157 at a concentration of 30 µM, including veratridine-elicited free radical production, mitochondrial depolarization, the release of cytochrome C, Na^+^ and Ca^2+^ currents (50–60%), and the elicited oscillations of cytosolic Ca^2+^ [100]. 

The benzothiazepine CGP37157 has been the selected drug to study the physiological role of NCLX in cell death/survival processes since the 1980’s [92]. However, its lack of selectivity, as it also blocks plasmalemmal NCX, voltage-gated calcium channels (VGCC), voltage-gated sodium channels, among other biological targets [83], compromises the relevance of the pharmacological results extracted from its pharmacological use. 

Recently, the inhibitory effects of the natural product curcumin on NCLX functionality, and its relevance in the context of colorectal cancer, has been reported [101]. Otherwise, this contribution stimulates the search in nature for new molecular drugs for neurodegenerative diseases possessing selective mechanisms of action and lacking undesired side effects, as shown in experiments with CGP37157. In the past, the discovery of huperazine A, a Chinese herbal medicine investigated for the treatment of Alzheimer’s disease, entailed a breakthrough in the study of new highly selective inhibitors of acetylcholinesterase, as an example [102]. 

**Figure 1:**
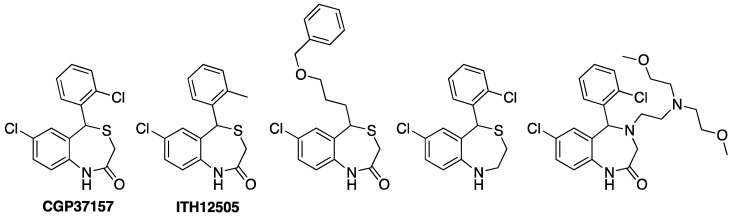
Chemical structures of CGP37157 and selected first generation analogues prepared, showing IC_50_ values to block NCLX in pancreatic cells between 2 and 7 nM [CGP37157, 1.4 nM)] [97].

Nonetheless, the closest isosteric analog of CGP37157, i.e., where the chlorine at C2′ was replaced by a methyl (second left, Figure 1, named ITH12505), was later evaluated using in vitro neuronal models of neurodegeneration [103], specifically chromaffin cells bovine adrenal medulla, the neuroblastoma SH-SY5Y cell line, and rat hippocampal slices. 

CGP37157 and its isosteric analog ITH12505 protected these in vitro neuronal models against toxic stimuli related to Ca^2+^ overload, i.e., bovine chromaffin cells subjected to the depolarizing stimulus elicited by veratridine 30 nM, SH-SY5Y cells subjected to high extracellular K^+^ concentration [104], and rat hippocampal slices acutely exposed to veratridine 30 nM or Glu 1 mM [103]. This fact is strengthened in experimental models where the calcium homeostasis modulator-1 channel (CALHM1) is present, as it has been described that CGP37157 blocked the wild type CALHM1 10 times better than the NCLX [105], becoming the first molecule reported to block that newly discovered ionic channel [106]. Hence, the neuroprotective actions elicited by CGP37157 could be ascribed to the NCLX blockade or effects on other targets. It is widely known that the mitigation of [Ca^2+^]_c_ overload leads to survival in neuronal models, while the complete abolition of Ca^2+^ efflux from mitochondria triggers mPTP opening and, in turn, apoptosis [107]. Thus, the rising question is whether the amenable reduction in the mitochondrial Ca^2+^ efflux elicited by CGP37157 contributes to its neuroprotective profile or, by contrast, counteracts it. A recent contribution tries to solve this dichotomy [108]. Using *C. elegans* as the animal model [109,110,111], it was observed that the administration of CGP37157 to nematodes at concentrations comparable to its IC_50_ to block NCLX induced an increase in their life span. Mutant *C. elegans* showing a defective complex I of the mitochondrial electron transport chain did not experiment with such lifespan extension. This would prove that the anti-aging properties of CGP37157 would involve, in part, a direct intervention on mitochondria functionality. Nevertheless, improved and more selective pharmacological tools keep being needed to better clarify the influence of Ca^2+^ fluxes between mitochondria and cytosol in the health state of neurons and other brain cells.

The first attempts to optimize CGP37157 pharmacological activity were reported in the early 2000’s. Pei and co-workers reported the synthesis of a set of CGP37157 derivatives that were evaluated as potential NCLX blockers in a pancreatic in vitro model, but none of them improved the blocking activity of CGP37157 [97] (Figure 3). By contrast, ITH12505 was more effective against oxidative stress-related damaging insults than CGP37157 [104], as SH-SY5Y neuroblastoma cells challenged with the stressor cocktail comprising rotenone 30 nM and oligomycin A 10 nM (R/O) were rescued by ITH12505 in a wide range of concentrations, but not by CGP37157. The R/O cocktail administration produces an interruption of the mitochondrial respiratory chain by combining rotenone and oligomycin A, which block complexes I and V, respectively. This induces the generation of free radicals and ATP synthesis impairment [112]. Thus, the pharmacological action of R/O evokes a resembling model of ROS generation originating in the mitochondria. In addition, the tissue viability of rat hippocampal slices was recovered by ITH12505 when compromising oxygen and glucose deprivation, and sub-sequent reoxygenation (OGD + redox), which is a neuroprotective effect not reproduced by CGP37157 [103]. Interestingly, CGP37157 exacerbates the cell death induced by R/O in a concentration-dependent manner, as shown in bovine chromaffin cells [100]. This particular observation would imply that CGP37157 fails to protect neuronal models against oxidative stress scenarios, which would limit their therapeutic potential.

**Figure 2:**
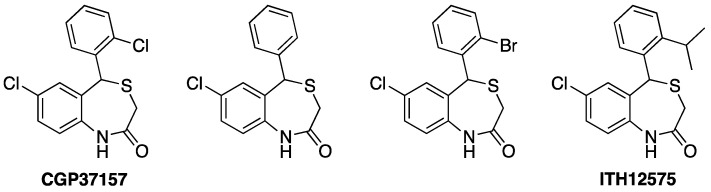
Chemical structures of CGP37157 and selected second generation analogues with NCLX blocking properties and neuroprotection [104].

Overall, the neuroprotective activity of ITH12505 against both Ca^2+^ overload and oxidative stress, this last being absent in CGP37157, could be ascribed to a more contributory effect of NCLX intervention in the neuroprotective profile of the new derivative. This rising hypothesis led to the chemical preparation of further analogs following modifications in the substitution at the pending phenyl ring [104] (Figure 2). In addition, CNS penetration was guaranteed, according to PAMPA experiments, which proved CGP37157 and its derivatives depict permeability values higher than 200 nm/s [103].

The planned chemical modifications focused on assessing the effect of the replacement of substituents at the pending aromatic ring and their spatial position. CGP37157 possesses a chlorine atom at *ortho* position. Thus, following a Topliss-like decision scheme, other halogens were probed (fluorine, bromine, two chlorines). These new derivatives presented a decent neuroprotective profile against both Ca^2+^ overload and oxidative stress in vitro neurodegeneration models, but they evoked a diminished NCX blocking effect, evaluated by mitochondria-targeted aequorin upon histamine stimulation [104]. Considering the good outcomes of the ortho-methylated ITH12505 (Figure 1, second left), CGP37157 analogs bearing alkoxy substituents were also prepared and tested. In these cases, the dramatic stereoelectronic change provoked a sharp reduction in the neuroprotection profile, as well as of the NCLX blockade. The logical step forward was probing the chain extension of the methyl group present in the hit ITH12505, leading to the synthesis of derivative ITH12575 (Figure 2, first right), which bears an iso-propyl group at a similar *ortho* position. This bulky hydrophobic substituent discriminates spatial conformations of the benzothiazepine scaffold, thus favoring target selection. This was confirmed in permeabilized HeLa cells challenged with a protocol defined by applying 10 mM Na^+^ to stimulate the mitochondrial Ca^2+^ release. In this situation, ITH12575 exerted about a two-fold fall in the clearance rate of mitochondrial Ca^2+^ compared with that by CGP37157 [104]. More importantly, ITH12575 scarcely affected Ca^2+^ currents in bovine chromaffin cells, thus indicating that it does not block VGCC, unlike CGP37157. Among other off-targets, ITH12575 lacks the huge blocking activity of the CALHM1 channel that CGP37157 shows [105]. Moreover, ITH12575 proved to be neuroprotectant in all the experimental models assayed, i.e., bovine chromaffin cells damaged with the depolarizing stimulus exacerbated by veratridine, SH-SY5Y neuroblastoma cells challenged by either the depolarizing stimulus defined by the increase in the extracellular concentration of K^+^ (70 mM) or the stressor cocktail R/O, and rat hippocampal slices subject to high glutamate exposure (1 mM), a widely used model of excitotoxicity.

Overall, a more potent and selective NCLX blocker has been reported, possessing improved neuroprotective properties. This statement should be sufficient to answer the recurrent debate on the eligibility of NCLX as a druggable pharmacological target to combat neurodegeneration. This does not mean that the full interruption, and therefore the huge accumulation of Ca^2+^ within mitochondria, was not harmful. Indeed, this is a question of recruiting mitochondria to manage, and in turn buffer, extreme Ca^2+^ elevations into the cytosol, because those pharmacological actions elicited by benzothiazepine derivatives of CGP37157 are simply downregulating the Ca^2+^ release rate back to cytosol. Finally, the optimized NCLX ligand ITH12575 is commercially available for its use in the plethora of physiological and pathological events where mitochondria play a key role.

**Figure 3:**
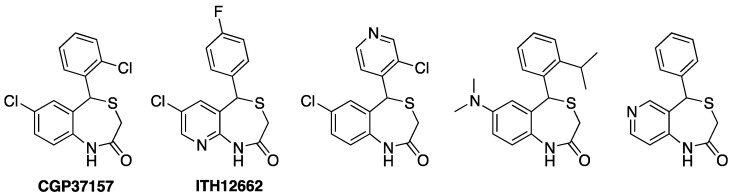
Chemical structures of CGP37157 (first left) and selected third generation analogues with improved pharmacokinetic properties [113,114].

Nevertheless, ITH12575 presents characteristics as a pharmacological tool that is not compatible with a potential therapeutic agent, as it is extremely lipophilic. The clog P of ITH12575 is around five, much higher than the value recommended to guarantee drug ability, which is about two, even if supposed to target the CNS. For this reason, further chemical optimization was focused on increasing polarity and the pharmacokinetic parameters of the benzothiazepine core, without compromising the potential pharmacodynamic properties. The selected strategy was the ring variation, namely turning the benzene-fused ring present in CGP37157, ITH12505 or ITH12575, into pyridine, to furnish pyridothiazepine derivatives (Figure 3) [113]. As an alternative, the replacement of the chlorine atom at C7 of the benzothiazepine ring with a dimethylamino group also afforded CGP37157 with low clog P [114]. 

**Figure 4:**
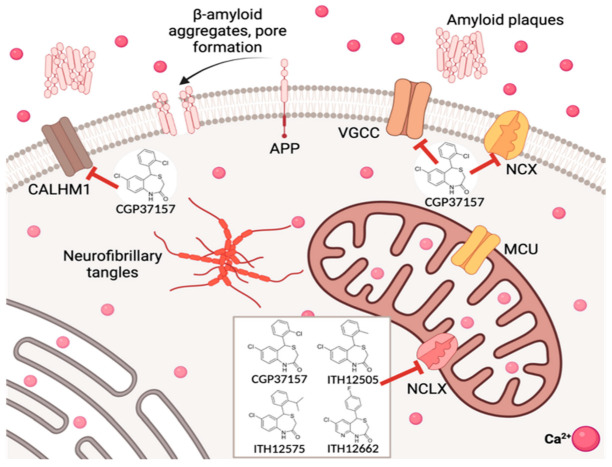
Biological targets of CGP37157 and derivatives that participate in cell Ca^2+^ regulation.

The replacement of the benzene by a pyridine-fused ring enhanced the global polarity of molecules, as demonstrated by comparing clog P among similar analogs of this series. The clog P lowering was also achieved by replacing the chlorine atom at C7 with a dimethylamino group, or the pending phenyl ring with pyridine. It is noteworthy that the pKa calculation ensured that the new pyridothiazepines and aminobenzothiazepines remain 95% neutral between pH 4 and 10, thus facilitating permeability. In general, the lowering in clog P was about 1-log unit. Experimentally, the aqueous solubility augmented noticeably, observed by UV-vis absorption spectra, as benzothiazepine derivatives collapsed at concentrations as low as 10 nM, while some pyridothiazepines reached the 0.3 mM concentration in PBS at physiological pH [90]. This amenable chemical modification would not only affect physicochemical parameters but would also allow the generation of new interactions with biological targets, by accepting H-bonds with the new aromatic nitrogen. Unfortunately, the disclosed pyridothiazepines exerted a differential neuroprotective profile. Some derivatives protected SH-SY5Y cells against calcium overload, and some against R/O, while others were better neuroprotectants in the depolarizing stimuli elicited by veratridine in bovine chromaffin cells. Overall, the best input was found in hippocampal slices subjected to excitotoxicity. However, again, compared with the family of benzothiazepines analogs to CGP37157, the incorporation of pyridine as a fused ring sensibly reduced neuroprotective capacity. As far as the assessment of the NCLX blockade, six out of ten pyridothazepines elevated the Ca^2+^ accumulative capacity of mitochondria upon histamine stimulation, monitoring maximal calcium peak, the area under the curve, and clearance rate, due to the mNCX blockade [113]. Indeed, in most cases, such figures were docile. However, in one case, that of the p-fluoroderivative ITH12662 (Figure 3, second left), the Ca^2+^ mobility through mitochondria experimented with a sharp reduction, underlining the maximal concentration of mitochondrial Ca^2+^ and the AUC of the [Ca^2+^]_m_. Hence, compound ITH16662 was selected as the best NCLX blocker of the family, with a similar potency to the hit CGP37157, slightly neuroprotectant, but with much better solubility than previous NCLX ligands. As was observed with ITH12575, it scarcely affected depolarizing-stimulated Ca^2+^ currents, thus confirming a lesser off-target behavior. Being a more promising therapeutic candidate, it has been extensively studied in several pharmacological models. For instance, ITH12662 was used to assess the role of mitochondria in the secretory catecholamine release mediated by cytosolic Ca^2+^ levels. The C57BL6J mice chromaffin cells stimulated with short ACh pulses manifested a secretory catecholamine release that, as expected, slowly decayed after repeated pulses [115]. The administration of ITH12662 at micromolar concentrations counteracted such decay, experimenting with a certain recovery of the amperometry-monitored catecholamine secretion. This observation was opposite to that found with CGP37157, which exacerbated the secretion decay. It was demonstrated by low-Ca^2+^ affinity, mitochondria-targeted aequorin that ITH12662 diminished mitochondrial Ca^2+^ efflux, but to a lesser extent than CGP37157. However, ITH12662 was proved to have the opposite regulatory actions on ACh-mediated catecholamine release to those found with CGP37157. It was hypothesized that ITH12662, by slightly reducing the release of Ca^2+^ from the mitochondria to cytosol without affecting other calcium transports, would be more efficiently buffering the Ca^2+^ levels necessary to refill and prepare ACh vesicles to pool, ready to be released by exocytosis. By contrast, the blockade of both nicotinic currents and K^+^ pulses performed by CGP37157 seemed to be detrimental for catecholamine release. This differential behavior underlines the high contribution of the VGCC blockade evoked by CGP37157 in its whole pharmacological effect on the cell physiology of excitable cells, which overpasses any intracellular action, such as its activity as an NCLX blocker. (Figure 4). By contrast, ITH12662, by lacking any effect on VGCC, influences secretory machinery maintenance unequivocally due to its down-regulating action of NCLX.

In summary, this contribution is a paradigmatic example of the critical role that the adequate selection of pharmacological tools has in the study of the physiological and pathological participation of receptors, channels, and other carrier proteins (Figure 4). The multipotent activity of some drugs, which is an increased value in drugs positioned for the treatment of several diseases such as neurodegenerative ones, could be the cause of scientific pitfalls in basic pharmacology, leading to wrong beliefs. Nowadays, there is still no agreement about whether the pharmacological intervention on NCLX exerts neuroprotection or triggers apoptotic signals. Moreover, advanced methodologies, such as the use of siRNA, have generated even more confusion, considering that they are an absolute manipulation of the mitochondrial physiology, obtaining the complete disruption of NCLX activity. The implementation of selective NCLX ligands, instead of the so-called promiscuous drugs such as CGP37157, in animal models of ND should help to obtain an agreement on the role of NCLX in both physiological and pathological scenarios.

## 5. Dephosphorylation Processes in Neuronal Damage: The Role of PP2A

The phosphorylation of hydroxylated amino acids is a post-transductional modification of proteins that plays a fundamental role in the majority of cell processes. This type of signaling capable of modulating cell physiology occurs through the incorporation or removal of phosphate groups at Ser, Thr or Tyr amino acids of the target proteins, which modifies their activity, 3D-shape, motion, and cell localization [116]. For this purpose, kinases, i.e., phosphorylating enzymes, but also phosphatases, i.e., dephosphorylating enzymes, act in a balanced fashion to finely determine the phosphorylation state of proteins. 

The relevance of the phosphorylation processes in cell physiology is corroborated by the battery of available drugs capable of inhibiting kinase enzymes, prescribed for a plethora of different human diseases, with cancer being highlighted. In the CNS, there are also a huge number of mechanisms where protein phosphorylation fulfills an essential function. For instance, the phosphorylation state of the microtubule-stabilizing tau protein influences the stability of the neurons’ architecture, as well as their plasticity [117]. When tau phosphorylation becomes disproportionate, this fine equilibrium is disrupted and, consequently, several associated pathologies rise, i.e., the so-called tauopathies [118]. 

In this dramatic neurodegenerative disease, the propagation of tau hyperphosphorylation derives from the loss of affinity for microtubules, and hence the stability of the neuronal cytoskeleton is compromised. In parallel, hyperphosphorylated tau acquires self-affinity, furnishing aberrant structures, the principal being the parallel helicoidal filaments (PHF), which subsequently form the so-called neurofibrillary tangles (NFT), one of the chief morphological hallmarks of AD in patients’ brains [119]. The relevance of all these pathological mechanisms gave rise to the protein tau hypothesis to explain the progression of AD [120], which has commonly been viewed as a rival of the A peptide hypothesis for the origin of AD [121]. In any case, an appreciable battery of kinase inhibitors has been probed in AD drugs to slow down the phosphorylation process, principally targeting the GSK-3β enzyme, but also inhibiting CdK-5, PKA, CaMKII or ERK1/2, among others [122]. This is by far one of the toughest challenges from a therapeutic point of view, considering that many kinase enzymes phosphorylate tau at various and different phosphorylation sites. For instance, the inhibition of a GSK-3β inhibitor, indeed the most investigated kinase inhibitor for AD, would not disrupt tau phosphorylation catalyzed by the other tau kinases. Curiously, the opposite dephosphorylation process is carried out mainly by a unique phosphatase, the phosphoprotein phosphatase 2A (PP2A) [123]. The Iqbal’s group reported that PP2A contributes to 70% of the whole phosphatase activity on tau, while others, such as PP1, PP2B, and PP5, are each responsible for about 10% of the tau dephosphorylation [124]. 

In addition, PP2A dephosphorylated key amino acids are implicated in dissociation from microtubules and self-aggregation. Interestingly, the phosphorylating activity of PP2A in AD patients’ brains is compromised [124]. These findings, among others, make PP2A an attractive biological target to impair the tau hyperphosphorylation evidenced in AD. Indeed, PP2A is one of the most abundant enzymes in eukaryotes and is ubiquitously expressed. It fulfills essential roles in development, proliferation, and cell death, as well as mobility and cytoskeleton dynamics [125]. PP2A is a key factor in cell cycle control and controls several signaling pathways. All these valuable functions mean that its depression and impairment develop various pathologies such as AD, Parkinson’s diseases, metabolic disorders, viral infections and, of note, many types of cancer.

However, the reality is that phosphatase enzymes are not biological targets selected for drug discovery projects, as happens for kinases. The rich therapeutic battery of compounds able to inhibit kinase enzymes has given rise to the so-called “kinoma”, where the hundreds of phosphorylating enzymes described are studied and shown in phylogenetic trees together with their pharmacology, and the therapeutic implications. There is a simplified belief that views phosphatase enzymes as very unspecific enzymes, so a therapeutic intervention would lead to many non-desired secondary effects [126]. Increasing evidence has proven that phosphatases, where PP2A prevails, are highly regulated enzymes, comprising a great variety of polymorphisms that discriminate activity, function, and cell localization.

For instance, the entire active PP2A is a trimer formed by: (a) a catalytic scaffold subunit called PP2A-A, having two possible polymorphisms, the Aα and the Aβ; (b) a subunit in charge of the enzymatic hydrolysis of phosphate esters from amino acids, called PP2A-C, that is also present in two possible polymorphisms, i.e., C′ and C″; and (c) a subunit with a regulatory role, called PP2A-B, which can be found in about 30 different polymorphisms, divided into four big subgroups (B, B′, B″, and B‴) [127,128]. Thus, by the combination of the diverse polymorphisms of each subunit, more than 70 types of PP2A have been found out so far. Therefore, more than the opinion that PP2A is not a reliable therapeutic target, the real fact is that drug discovery has a tremendous challenge in the next years, which is the search for selective drugs capable of interacting selectively with some of the many existing PP2A subtypes. In addition, PP2A activity, like other Ser/Thr phosphatases, is tremendously regulated [129]. 

Right after the synthesis of the catalytic PP2A-C subunit, it is bound to α4 and PP2A-methylesterase-1 (PME-1) enzymes that prevent PP2A activation [130]. This last enzyme impedes methylation at the PP2A terminal carboxy in Leu309. The entire activation of the PP2A trimer, where the three subunits A (structural), B (regulatory), and C (catalytic) are connected, is achieved by the actions of several enzymes. The phosphotyrosyl phosphatase activator (PTPA) is a Tyr phosphatase that dephosphorylates PP2A and favors an adequate conformation for the leucine carboxyl methyltransferase-1 (LCMT1) to introduce a methyl group at the terminal carboxy of Leu309 [131]. These post-translational modifications augment the affinity of the dimer AC by the regulatory B subunit. By contrast, phosphorylation at Tyr307 weakens the trimer stability and, in turn, PP2A activity is diminished [129]. 

Regulatory mechanisms for PP2A are reinforced by the action of several peptides that operate as endogenous inhibitors, such as I1PP2A and I2PP2A (also called SET [132] and CIP2A [133]), among others. They mostly interact with the catalytic PP2A-C, impairing its enzymatic action over phosphoprotein substrates. Their role is so important as the pathological situations where PP2A activity is compromised, for instance in several types of cancer or neurodegenerative diseases, are mostly due to an overexpression or hyperactivity of some of these PP2A endogenous inhibitors [134,135]. In addition, several mutations in PP2A expression have been correlated with a deficient activity, which have been observed in different pathologies [136].

## 6. PP2A-Activating Drugs

Noticing that PP2A fulfills significant roles in a plethora of physiological functions within cells, and its depression has been documented in a huge amount of diverse human diseases, most drug discovery programs have aimed at finding PP2A-activating drugs (PADs) [137]. This implies another hurdle in the way, as the design and development of enzyme inhibitors are much easier than for activators. A direct activator should trigger or enhance enzymatic activity upon interaction, but the common consequence of any drug binding is, by contrast, enzymatic inactivation. This has slowed down the finding of new PADs. Otherwise, a reliable approach chosen by many research laboratories has been the design of ligands for the regulatory enzymes of PP2A, or its endogenous inhibitors. These drugs belong to the first family of PADs stated by Stoke and co-workers, namely the inhibitors of inhibitory actions [138]. 

**Figure 5:**
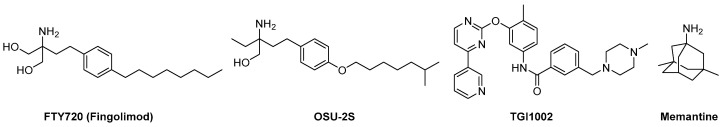
Chemical structures of PP2A-activating drugs (PADs) that act through inhibiting SET.

The most extended family is that of SET inhibitors (Figure 5), among which the ceramides derivatives prevail. Indeed, the most studied PAD in the clinic is the sphingosine analog fingolimod (or FTY-720) (Figure 5). Besides its immunosuppressant effect derived from an agonist activity of the sphingosine-1-phosphate receptor, fingolimod interacts with SET at its PP2A-C binding site, provoking dissociation of the PP2A/SET complex and the subsequent translocation of SET to the nucleus [139]. Fingolimod is used for the treatment of multiple sclerosis and is being investigated for the treatment of several malignancies [140]. Other compounds capable of disrupting the PP2A/SET complex are the apoEpeptidomimetics COG112 and OP449 [141], and the low-molecular-weight drug TGI1002 (Figure 5) [142]. Of note is the action exerted by the NMDA antagonist memantine (Figure 5), which avoided the SET translocation from the nucleus to cytosol, independently of its antiglutamatergic profile, as other NMDA antagonists lacked this behavior [143]. 

**Figure 6:**
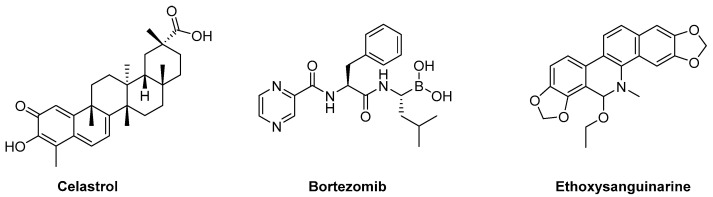
Selected PADs acting through the inhibition of CIP2A.

Several drugs have been claimed to inhibit CIP2A (Figure 6). The pentacyclic terpenoid celastrol traps CIP2A and forms a complex directed to the ubiquitin-marked proteasome degradation [144]. Bortezomib, approved for several cancers, seems to down-regulate the posttranslational expression of CIP2A [145]. Other CIP2A modulating drugs are etoxisanguinarine [146] and niclosamide [147]. 

**Figure 7:**
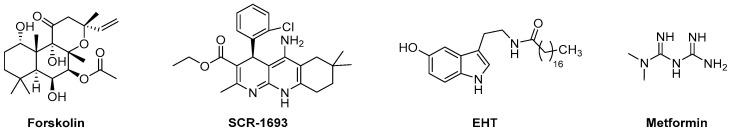
Chemical structures of PADs acting on the post-transductional processing of PP2A.

The second family of PP2A activators comprises drugs that affect the post-translational regulatory mechanisms of PP2A. The phosphorylation of PP2A, mainly at Thr304 or Tyr307, hinders the formation of the complete PP2A trimer. In this context, forskoline (Figure 7), independently from adenylate cyclase activation, reduces the phosphorylation rate of PP2A at Tyr307 [148]. The tacrine-dihydropyridine SCR-1693 reduced phosphorylation at Tyr307 by a mechanism still elusive. On the other hand, the coffee alkaloid N-eicosanoilserotonine (EHT) inhibits the demethylating enzyme PME-1, protecting the PP2A activity. Because of its dephosphorylating effect on tau protein and down-regulation of 2A expression, it has been studied to mitigate aging-dependent cognitive disorders [149]. Another group of PADs is the one affecting PP2A degradation. For instance, the antidiabetic drug metformin has been reported to interfere with the MID/α4 complex necessary to degrade PP2A via proteasome [150] (Figure 7). 

**Figure 8:**
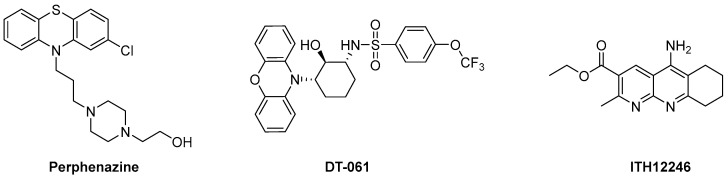
Chemical structure of selected direct PADs.

Otherwise, there are many other drugs described to exert an up-regulatory action on PP2A through unknown action mechanisms. Such is the case of melatonin, whose pharmacodynamic actions include counteracting the glutamate-elicited excitotoxicity by mitigating the under-expression of the PP2A-B subunit [151]. In a recent review, we have proposed that melatonin could directly interact with PP2A at the intersection between the A and C subunits [152]. This last mechanism indeed comprises the last family of PADs herein reviewed, defined by compounds directly interacting with PP2A. In general, the real mechanism by which they produce PP2A activation is not completely understood, highlighting the difficulty of displaying an enzyme–drug interaction lacking inhibitory actions. Basic amines such as protamine or polylysine, positively charged at physiological media, promoted PP2A activity through direct interaction with PP2A-C [153]. The drug family of phenothiazines has been an object of studies as PP2A direct activators, corroborated by mass spectrometry-assisted proteomic studies, where the drug perphenazine has been underlined (Figure 8), which seemed to bind the PP2A A subunit [154]. Another family of direct PADs is that named SMAP (small-molecule activator of PP2A), where the tricyclic sulfonamide DT-061 stands out (Figure 8) as it diminished tumor progression in KRAS-mutated lung cancer, presumably by interacting with the polymorphism of the PP2A-A subunit [155]. In this context, our first contribution to the discovery of new direct PADs emerged from the study of a family of anticholinesterasics related to tacrine that presented potent and selective AChE inhibition [156]. They displayed proven neuroprotective properties against oxidative stress-induced neurodegeneration in SH-SY5Y neuroblastoma cells, but not against okadaic acid (OA), except with one compound, which we have named ITH12246 (Figure 8).

The marine toxin OA represents a reliable model of neurodegeneration with its origin in tau hyperphosphorylation, due to it producing a huge and highly selective inhibition of PP2A [157]. Considering the critical role of PP2A as a tumor suppressor, OA has also been used to resemble cancer proliferation originating in PP2A underactivity [158]. Otherwise, in the clinic, OA is responsible for diarrheic shellfish poisoning, provoked by the intake of mollusks contaminated with some microalgae [159]. This is due to the severe inhibition of the cellular dephosphorylation at the bowel. Interestingly, some individuals evidence a transitory loss of memory. This is due to the pathological manifestations that OA evokes in the CNS that give rise to neurodegeneration, the most important being tau hyperphosphorylation conducting to the so-called tauopathies. Thus, the administration of OA to neuronal cultures or animal models resembles AD progression [160]. 

We used OA to induce tauopathy and therefore neurodegeneration in SH-SY5Y neuroblastoma cells. Among the family of tacrine derivatives disclosed, only ITH12246 dissipated the OA-associated loss of cell viability in a statistically significant manner. Moreover, ITH12246 reduced neurotoxicity exacerbated by OA in the same experimental model and abolished the loss of tissue viability of rat hippocampal slices challenged with oxygen and glucose deprivation followed by reoxygenation, or by the excitotoxicity developed by high glutamate [161]. Realizing that all these neuroprotective properties should not be fully ascribed to AChE inhibition, and with the peculiar counteracting effect over OA neurotoxicity in mind, we wondered whether ITH12246 could promote PP2A enzymatic activity somehow. At first glance, in vitro malachite experiments confirmed that ITH12246 recovered PP2A activity compromised by OA [156]. Confirming this, in an in vitro test with only PP2A present, purified by immunoprecipitation, we found that the neuroprotective action of ITH12246 against glutamate in hippocampal slices was recruiting PP2A, as the presence of OA abolished any neuroprotection carried out by the new PAD [161]. Considering that PP2A down-regulates NMDA-sensitive glutamate receptors, as it dephosphorylates the NMDA receptor NR1 subunit [162], thus accelerating the coupled ion channel desensitization, ITH12246 would abrogate the glutamate-evoked neuronal damage by stimulating the PP2A-addressed NMDA receptor desensitization. Using computational techniques, we observed that ITH12246 presented structural and electronic similarities with the central moiety of OA, which is not directly implicated in PP2A inhibition, but only in increasing enzyme affinity. Blind molecular docking studies corroborated that ITH12246 could interact with PP2A in the same binding site of that targeted by the C19–C37 fragment of OA [156]. This finding forged the hypothesis that ligands mimicking the central moieties of the highly potent and selective inhibitor OA could have missed the inhibitory potential over PP2A, but maintain part of the interactive capacity at amino acids not directly implicated in the phosphate ester hydrolysis on the phosphoprotein substrate, and that binding could, in turn, hinder the access of endogenous inhibitors, such as SET or CIP2A. Hence, the final consequence would be the pharmacological protection of the phosphatase activity elicited by PP2A. Indeed, the exposure of ITH12246 to SH-SY5Y neuroblastoma cells triggers a global neuroprotection signal, as demonstrated by the modulation of gene expression monitored by the QI-AGEN-based analysis tool Ingenuity [163]. Several genes related to antioxidant enzymatic machinery were overexpressed upon ITH12246 administration, as important as HO-1, GSR, SQSTM1, and TRXT1. CDKN1A, which expresses P21, while BCLXL genes are the most highlighted overexpressed genes related to reduction in cell apoptosis. In addition, the Wnt family cascade, implicated in cell senescence regulation, was also induced, with the Wnt6 gene induced preferentially. This would also favor the ITH12446 capacity as a wide spectrum neuroprotectant.

**Figure 9:**
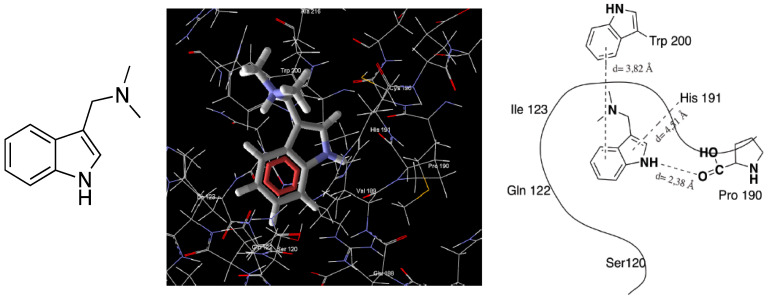
Structure of gramine and its pose at the PP2A-C binding site (Right, schematic view).

Overall, this new PAD offers neuroprotection against toxic stimuli related to PP2A depression, whose principal hallmarks are tau hyperphosphorylation and extended excitotoxicity. Using ITH12246 as an innovative drug to treat neurodegeneration, we probed its ability to mitigate cerebral damage in two in vivo models that resemble neurodegeneration, specifically in mice suffering a scopolamine-induced loss of memory, as a model of AD, and mice subjected to photothrombosis, as a model of focal cerebral ischemia [161]. The anticholinergic drug scopolamine generated a drop in the individuals’ memory index that was softened by 10 mg/kg of ITH12246, in a similar fashion to that exerted by the anti-cholinesterase AD drug galantamine, measured by an object placement test. Regarding the photothrombosis experiments, a four-fold lower concentration of ITH12246 was able to reduce the infarct volume observed in the mice subjected to the focal ischemia induction by 60% [163]. All these outcomes reinforced our idea of further delving into the finding of new PP2A ligands that promoted its phosphatase activity through direct interaction. However, the fact that only one chemical entity showed a pro-PP2A profile among a narrow family of 1,8-naphthyridines, which differed from each other in very few tiny structural modifications, pushed us to search for this non-conventional pharmacological activity in very different chemical space. During the search for new active compounds capable of preventing PP2A inhibition, we paid attention to the natural alkaloid gramine (Figure 9), extracted from the family of Poaceae plants [164]. In a regular screening of our chemical library, gramine showed a proven huge and robust neuroprotective effect against the OA toxicity in neuroblastoma cells, tested from nanomolar concentrations up to the high micromolar. In addition, predictive computational analysis pointed out some possible poses of gramine at the PP2A-C binding site (Figure 9, unpublished results). However, gramine also shows several issues, as it suffers rapid metabolization and derives in some toxic events. For this reason, we embarked on a profound chemical design of gramine derivatives with enhanced chemical interactions with PP2A, increased metabolic stability, and reduced the rate of adverse effects.

**Figure 10:**
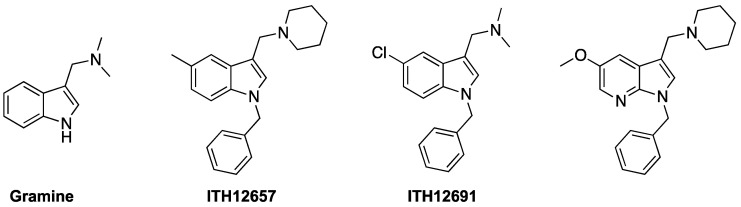
Highlighted gramine derivatives with important PAD activity.

**Figure 11:**
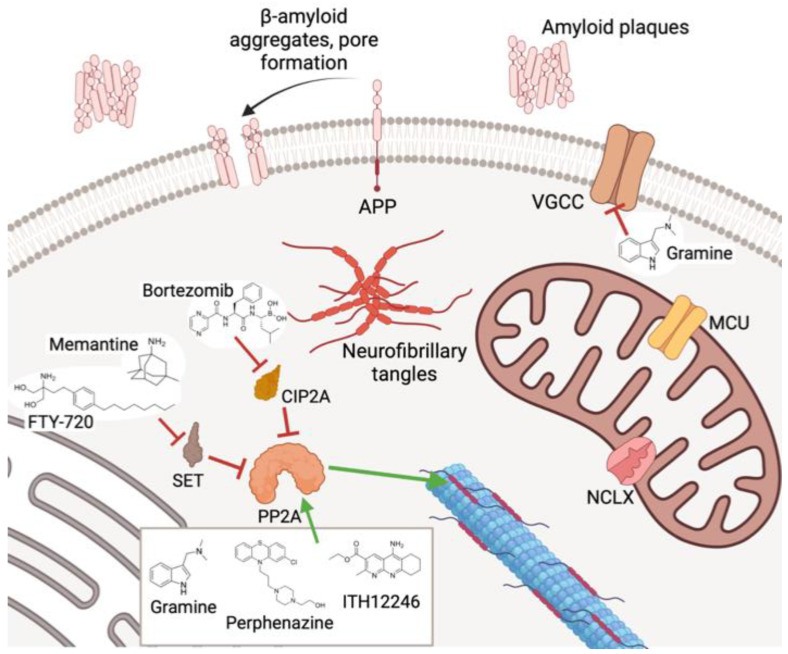
Pharmacological modulation of PP2A activity by different strategies.

According to the computational prediction based on molecular docking, the presence of an ionizable tertiary amine was preserved to favor interaction with PP2A, backed by the PP2A interactive behavior of some basic amines such as protamine. The aromatic indole scaffold presented in gramine would be highly stabilized (reducing the rate of the retro-Mannich reaction) if the aromatic nitrogen was alkylated. Thus, several substitutions were challenged, for instance, benzyl, n-butyl, propargyl, and so on, which in turn could generate an additional hydrophobic interaction with neutral amino acids present in the binding site of PP2A-C, far from the phosphate ester hydrolysis catalytic triad [165]. Finally, substitution at C5 of the indole core was diversely probed, incorporating methyl, halogens, or no substitution, to assess the different interactive possibilities with the enzyme [166]. Three dozen gramine derivatives were prepared by this straightforward method, and the ionized formulation facilitated drug solubility and their testing in further in vivo models. Their ability to recover phosphatase activity was monitored by the pNPP method in SH-SY5Y cultures subjected to OA. They showed a docile increase in phosphatase activity. In parallel, using patch-clamp recordings and fluorescence experiments with the Ca^2+^-sensitive probe Fluo-4, we appreciate an important blocking effect on high K^+^-evoked depolarization, whereby we hypothesized these compounds were acting as VGCC antagonists, too. In most cases, those compounds capable of performing such a dual pharmacological, pro-PP2A and VGCC blockade, illustrated a very noticeable neuroprotective profile in the experimental in vitro model of the SH-SY5Y cells exposed to either OA or depolarizing stimuli such as veratridine. The best compounds of the series confirmed their neuroprotective capability in hippocampal slices challenged with glutamate. Molecular docking predicted that the new gramine derivatives posed in a binding pocket of PP2A-C defined by the amino acids His191, Ser120, Trp200, and Ile123, residues far from the catalytic site, but that it contributes to drug affinity. In addition, Western blot analyses proved that the most highlighted compounds such as ITH12657 or the chlorinated derivative ITH12691 reduced the OA-induced tau phosphorylation rate [165,166] (Figure 10). Because of the PAD role of ITH12657, together with its important VGCC activity, preferentially to the P/Q subtype, it is currently under investigation for the treatment of neuropathic pain and retinopathies, indications where PP2A and the VGCC play a chief role (Figure 11). As far as ITH12691 is concerned, its global pharmacological profile has addressed the characterization to appraise its potential in neurodegeneration, as preliminary experiments anticipate that it could augment the discrimination index of LPS-subjected mice, according to the object-location memory task.

Nevertheless, the fact that this new family of real PAD possessed another significant pharmacological activity may be relevant from a therapeutic point of view, where the multitarget approach for the treatment of neurodegenerative diseases is a highly reliable strategy. However, from a pharmacological point of view, it does not clarify how the recovery of the PP2A activity contributed to the whole neuroprotective profile (Figure 11). For this reason, finding a selective PAD that lacks off-target poly-pharmacology, though that was beneficial for the disease, turns into a priority. Interestingly, we prepared a new analogous family of gramine derivative defined by a ring variation in parallel, similar to what we conducted for the development of CGP37157 derivatives. Thus, the benzene-fused ring present in the indole core of gramine derivatives was replaced by pyridine, furnishing 7-azaindoles derivatives (Figure 10, first right) [166]. Firstly, this is an attractive chemical modification that increased the polarity of chemical entities, as corroborated by the resulting averaged clog P of the new family, significantly diminished compared with those from the indole family, making this new family a more druggable series of potential drugs [166]. It is noteworthy that this new family presented similar characteristics to PADs, but lacked any activity over VGCC. Despite this loss, the new family of 7-azaindoles analogs to gramine is, therefore, more selective and does not possess so many off-target issues. Thus, it becomes an eligible tool to validate PP2A as a therapeutic target for neurodegeneration.

The most recent step forward has been the incorporation of 7-azaindoles into hybrid systems based on clay minerals, to facilitate oral administration and their controlled release. We tested several nanocarriers, such as montmorillonite and halloysite nanotubes, but the real controlled release in systems simulating the gastro-intestinal tract was achieved by encapsulating the hybrid material into a biopolymer matrix composed of alginate and zein. Thus, the biononcomposite complexes encapsulating the 7-azaindole/clay hybrids enhance the pharmacokinetic features of this series of gramine azaderivates [167]. Otherwise, attempts to increase pharmacodynamic outcomes within these types of derivatives have been challenging; these types of indole alkaloid-based drugs have only promoted PP2A activity smoothly. We realized that the design of more potent PADs should be rationalized by a computer-aided drug design. Under the premises of the increased affinity elicited by the structural motifs of OA that are not directly implicated in PP2A inhibition, we decided to design analogs to the central fragment of OA, which is limited by C19 and C27 carbons. It is well known that this fragment interacts specifically with the hydrophobic region comprising Trp200, His191, Gln122 and Ile123. 

Hence, small analogs of the C19–C27 fragment should interact similarly to this allosteric pocket. As proof of concept of this new hypothesis, we have recently tested an earlier intermediate, which we have named ITH12680, that already possesses structural similarity to the central fragment of OA [168]. 

Despite its simplicity, ITH12680 generated an appreciable recovery of PP2A activity, measured by a more accurate malachite green method, using several polymorphisms of PP2A isolated by pull-down protocols [168]. The PAD activity of ITH12680 was confirmed by Western blot analyses of the expression of several phosphoprotein substrates of PP2A, such as Akt, ERK1/2, or Bax. This first proof-of-concept research has prompted us to design new OA central fragment analogs for the development of PADs with a focus on the treatment of neurodegenerative diseases, where PP2A malfunction has been reported as a common event.

## 7. Concluding Remarks and Future Perspectives

Many cellular homeostatic functions, ranging from energy generation to cell death and necrosis, are regulated by calcium influx into the mitochondria. The new molecular identification of the NCLX, mitochondrial calcium uniporter complex has offered vital insight into the function mitochondrial calcium influx plays in energy production under an elevating workload, and in the progression of diseases such as neurological disorders. The present review discussed the necessity of understanding the processes that regulate mitochondrial calcium influx and activity in neurodegeneration. PP2A activity is downregulated in tumors as a proven tumor suppressor, and reactivation of the protein can cause cancer cells to die. Reduced PP2A activity promotes tau hyperphosphorylation and Aβ production in the brains of AD patients. Upstream mechanisms that inactivate PP2A in illnesses have not been fully explained, and more research will aid in the refinement and development of new PP2A-targeted drugs for the treatment of ND (Figure 11). There is a wealth of information that PP2A regulates cancer and inflammation, and a lot of evidence is available that bioactive compounds from natural sources may activate PP2A and have therapeutic benefits. Clinical studies of these substances are most likely to initiate with anti-inflammatory and immune-modulatory activity where the evidence base is greatest, and the risk–benefit ratio is most favorable to research and clinical trials. When such studies are conducted, it would be prudent to track their effectiveness in combating neuronal damage. If the safety profiles of PADs are proven to be satisfactory, there is a case to be made for starting clinical studies in the setting of neurological diseases as quickly as possible. It is worthwhile mentioning that neurodegenerative diseases are multifactorial. Hence, several physiopathological mechanisms converge to favor and accelerate neuronal damage. This scenario has led to the idea that interacting with two or more pharmacological targets implicated in the progression of the disease, either by a combination therapy or by the so-called multi-targeted-directed ligands (MTDL) strategy, could be beneficious to counteract neurodegeneration. Indeed, there are several examples where this hypothesis has been implemented. For instance, AD patients are usually treated with a combination of the NMDA receptor blocker memantine and an anticholinesterasic, e.g., rivastigmine. As far as the MTDL strategy is concerned, there is a plethora of chemical entities described in the literature designed to act in a multitarget fashion to combat neurodegenerative diseases. Most of these studies have centered on very few targets, such as cholinesterase or MAO enzymes. We think it is time to deepen into other new biological targets such as those reviewed herein. However, as we emphasize above, to fully validate a MTDL strategy based on these new targets, it is necessary to prove that its selective intervention induces a neuroprotecting signal.
